# Fractal grid-induced turbulence strength characterization via piezoelectric thin-film flapping velocimetry

**DOI:** 10.1038/s41598-021-02680-7

**Published:** 2021-12-02

**Authors:** Ted Sian Lee, Ean Hin Ooi, Wei Sea Chang, Ji Jinn Foo

**Affiliations:** Mechanical Engineering Department, School of Engineering, Monash University Malaysia, Subang Jaya, Malaysia

**Keywords:** Energy science and technology, Engineering

## Abstract

The centerline streamwise and cross-sectional (*x*/*D*_*h*_ = 0.425) turbulence characteristics of a 2D planar space-filling square-fractal-grid (SFG) composed of self-similar patterns superimposed at multiple length-scales is experimentally unveiled via piezoelectric thin-film flapping velocimetry (PTFV). The fluid–structure-interaction between a flexible piezoelectric thin-film and SFG-generated turbulent flow at *Re*_*Dh*_ = 4.1 × 10^4^ is investigated by analysis of the thin-film’s mechanical response. Measurements of the thin-film-tip deflection *δ* and induced voltage *V* demonstrate increasing flow fluctuation strength in the turbulence generation region, followed by rapid decay further downstream of the SFG. Interestingly, SFG-induced turbulence enables the generation of maximum centerline thin-film’s response (*V*_*rms*_, *δ*_*rms*_) and millinewton turbulence-forcing (turbulence-induced excitation force acting on the thin-film) *F*_*rms*_ which are respectively, 7× and 2× larger than the classical square-regular-grid of similar blockage ratio. The low frequency, large-scale energy-containing eddies at SFG’s central opening plays a critical role in driving the thin-film vibration. Most importantly, the SFG-generated turbulence at (*y*/*T* = 0.106, *z*/*T* = 0.125) away from the centerline allows equivalent mechanical characteristics of turbulence generation and decay, with peak of 1.9× nearer from grid. In short, PTFV provides a unique expression of the SFG-generated turbulence, of which, the equivalent turbulence length-scale and induced-forcing deduced could aid in deciphering the flow dynamics for effective turbulence management.

## Introduction

Fractal grid has received an increasing amount of interest recently as a turbulence generator to enhance fluid mixing and convective heat transfer^1–4^. Fractal is a geometrical structure that repeats itself and diminishes in size, forming a complex pattern of several iterations^5,6^. A square fractal grid (SFG) restructures the upstream flow to generate more intermittent vorticity field, higher vorticities and higher turbulence intensities when compared to classical regular grid (RG) of similar or smaller blockage ratio^7^. A piezoelectric transducer converts mechanical vibration into AC electrical signal through piezoelectric effect. Despite possessing high mechanical strength, a piezoelectric thin-film has a wide frequency range and is highly responsive to turbulent flow^8^. When immersed in a fractal grid-induced flow domain, the random vortical structures shed from the grid bars will impart a mechanical strain on the piezoelectric to generate electrical charges as a function of fluid fluctuating properties.

Significant work has been carried out lately to investigate the interplays between fluid flow properties and random excitation force against piezoelectric’s structural and electrical responses. Akaydin et al.^9^ theoretically estimated the piezoelectric tip deflection from open-circuit voltage measurement to characterize the interaction between piezoelectric beam and turbulent eddies in the wake of cylinder based on the statistical properties of the voltage and deflection’s local extremes. The piezoelectric tip deflection at various free stream velocities and distances from the test section wall was measured using a strain-gage to study the fluid–structure interaction in turbulent boundary layers^10^. Forces measurement and theoretical analysis demonstrated the dependence of turbulence-induced excitation force upon the local mean velocity, turbulence intensity and relative size of the beam length to the integral length scale of turbulence. Experimental work^11^ showed that the force acting on the piezoelectric beam depends on the velocity and length scales of turbulence. The average power harvested can be modelled as a power-law with respect to the piezoelectric’s distance from the turbulator which closely follows the decay rate of turbulence kinetic energy. A frequency response model^12^ was established to compute the drag force applied to a piezoelectric vibration sensor from the experimentally recorded voltage signal. The force amplitude was reported to be linearly correlated with the Laser Doppler Anemometry-measured kinetic energy fluctuation intensity rather than the velocity fluctuation^13^.

In spite of the extensive research conducted over the past decade to study the behaviour of piezoelectric thin-film in highly unsteady flow, the majority of them focused on evaluating the piezoelectric performance in turbulent boundary layers^10,14^, cylinder^9,14,15^ and passive rectangular grid-induced flow^11,16,17^ for energy harvesting purposes. To date, the characterization of fractal grid-induced turbulence based upon the direct interaction of the flow with a piezoelectric thin-film has not been given much consideration in literature. Although recent experimental studies^18–20^ were conducted to investigate the effects of grid-beam distance on the electrical power extracted by a single and two side-by-side piezoelectric beams from fractal grid-induced turbulence, the piezoelectric output profile over the grid’s cross-section remains obscure. Moreover, the fractal grid-generated turbulence force acting on a flexible piezoelectric thin-film has yet to be unravelled.

Hence, the current study aims to experimentally unveil the interaction between a flexible piezoelectric thin-film and fractal grid-induced fluid flow by means of analysing the thin-film’s physical response towards turbulent flow dynamic. In particular, the expression of local equivalent fluid flow undulation and the corresponding turbulence forcing (turbulence-induced excitation force acting on the thin-film) via an in-house established system, namely, the piezoelectric thin-film flapping velocimetry (PTFV) to comprehend the fundamental turbulence and the strength of grid-generated flow. In the present work, a polyvinylidene fluoride (PVDF) piezoelectric cantilever beam is placed at different lateral positions and centerline streamwise distances leeward of SFG and RG to measure the film’s tip deflection and voltage response. A detailed exploration of the various equivalent turbulence statistics determined from the film undulation allows the mechanical characteristics of the grid-induced cross-sectional and centerline streamwise turbulent flow structures to be effectively expressed.

## Methods

### Experimental setup

**Figure 1:**
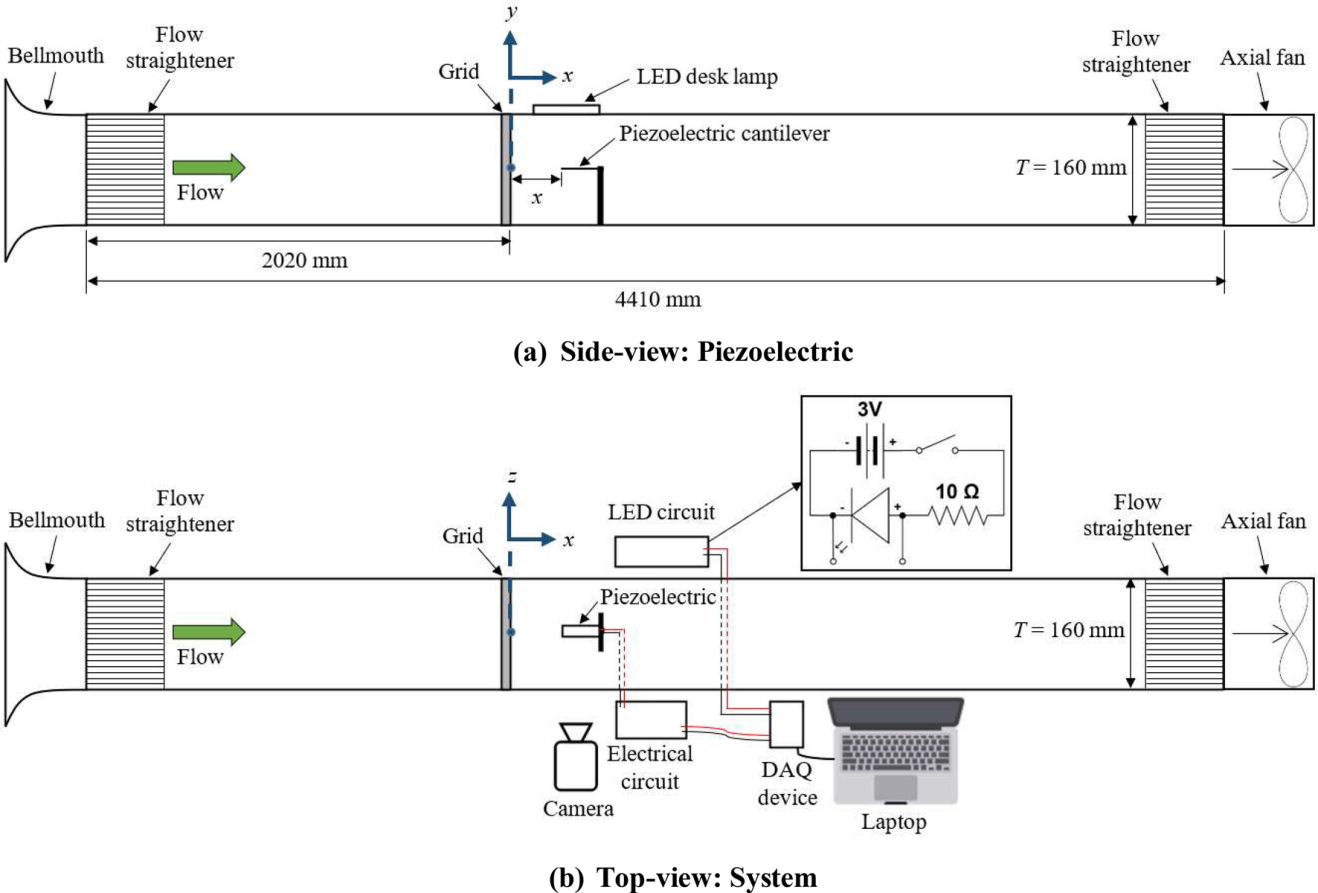
Schematic of experimental setup: (**a**) Side-view and (**b**) top-view.

**Figure 2:**
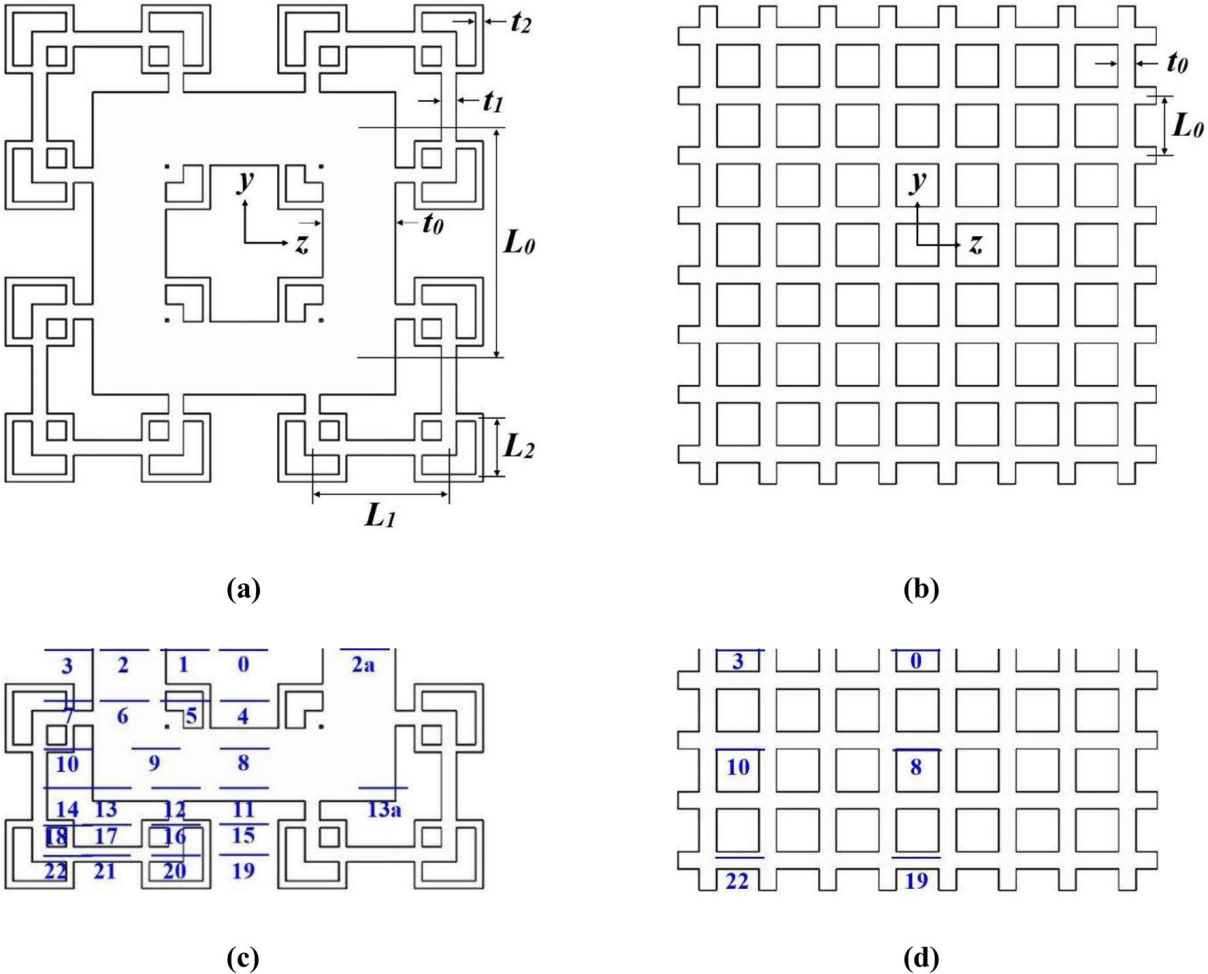
Schematic of (**a**) (*N* = 3, *t*_*r*_ = 9.77) SFG^4^ and (**b**) RG. Positions *P*_*0*_ to *P*_*22*_ indicate the individual allocation of piezoelectric thin-film in the lee of (**c**) SFG and (**d**) RG.

A schematic of the experimental setup is presented in Fig. 1. The *T* = 0.16 m wide and 4.41 m long square wind tunnel is constructed from 10 mm thick transparent acrylic sheets with a bellmouth attached to the inlet. Air is drawn into the channel by an axial fan (Kruger MTD200, SG) at a fixed centerline inlet velocity *v*_*in*_ = 4 m/s with hydraulic diameter-based flow Reynolds number *Re*_*Dh*_ = 4.1 × 10^4^. Flow straighteners are placed at the inlet right after the bellmouth and in front of the fan at the outlet. A 10 mm thick 2D planar space-filling SFG with fractal iteration *N* = 3 is inserted at 2.02 m from the inlet. The geometry of the fractal insert is the same as that numerically optimized by Hoi et al.^4^ in their numerical study, with thickness ratio *t*_*r*_ = 9.76 (*t*_*r*_ = *t*_*0*_/*t*_*2*_*,* see Fig. 2a) and blockage ratio (ratio of area covered by grid over channel cross-section): *σ* = 0.493. The RG employed in this study has equal thickness and *σ* as the SFG. Figure 2a,b show the schematic of the inserts with their geometrical parameters listed in Table 1. Note that $x_{*}$ denotes the wake-interaction length scale representing the location on the centerline where the wakes resulting from the largest grid bars interact, and can be expressed as^21^,

1 $$x_{*} = \frac{{L_{0}^{2} }}{{t_{0} }}$$

where *L*_*0*_ and *t*_*0*_ are the length and width of the largest grid bar, respectively.

**Table 1:** Geometrical parameters of SFG and RG.

Parameter	SFG	RG
*σ*	0.493	0.492
*L*_*0*_ (mm)	77.0	20.0
*L*_*1*_ (mm)	45.7	
*L*_*2*_ (mm)	20.4	
*t*_*0*_ (mm)	24.4	5.75
*t*_*1*_ (mm)	5.00	
*t*_*2*_ (mm)	2.50	
$x_{*}$ (mm)	243.0	69.6

**Figure 3:**
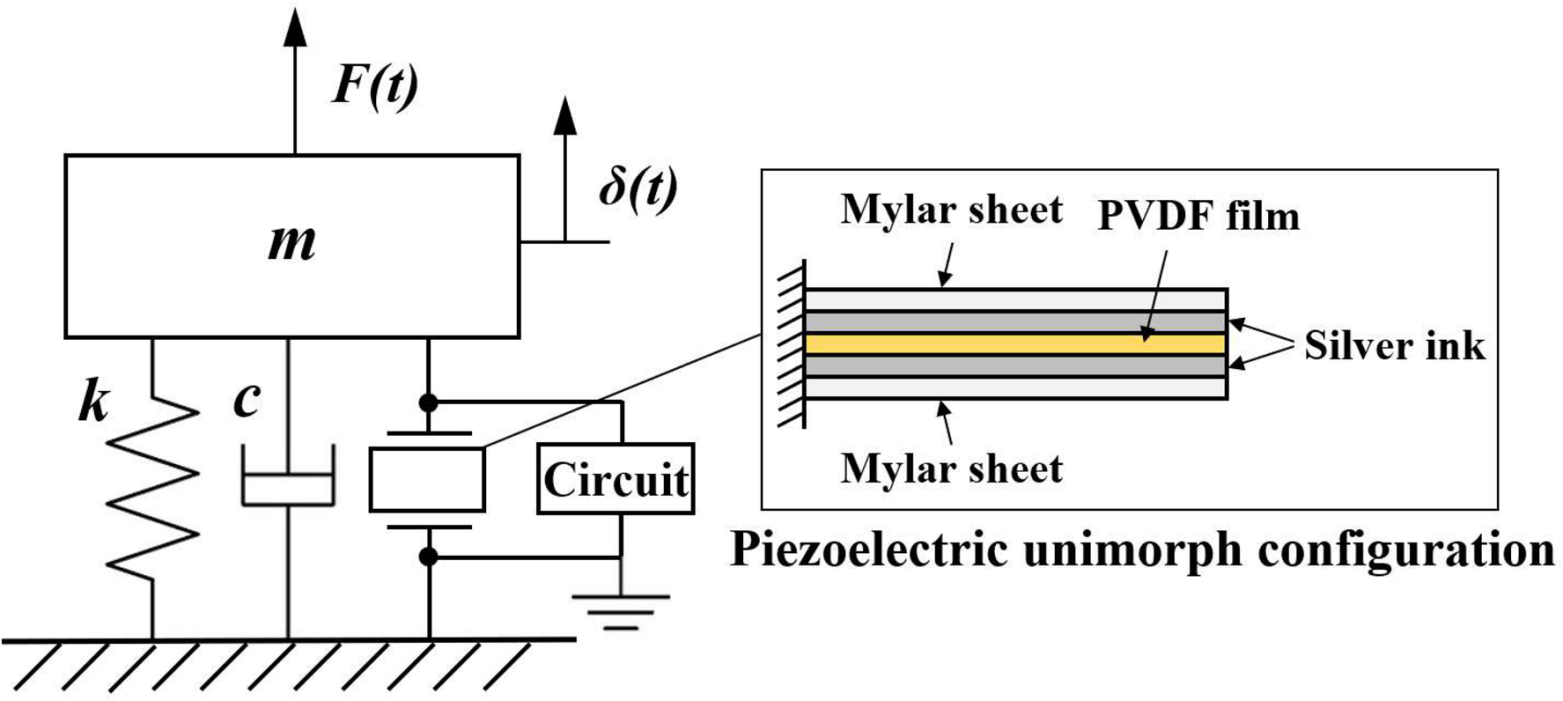
Schematic of the SDOF piezoelectric cantilever beam model subjected to a time varying arbitrary force *F*(*t*).

The unimorph piezoelectric thin-film (TE Connectivity LDT1-028 K, CH) is composed of a PVDF film element with silver ink screen printed electrodes laminated to a Mylar sheet (see Fig. 3). The properties of the thin-film^8,16,22^ are summarized in Table 2. The thin-film is clamped onto a 3D printed holder and placed parallel to the flow with its output connected to a 10 MΩ external electrical load. The thin-film edge is fluorescently labelled and illuminated by an LED light source. The piezoelectric voltage signal is sampled and digitised at a sampling rate of 10^3^ Hz using a data acquisition device (LabJack U3-HV, US) with a voltage resolution of 4.88 mV/bit. Note that the voltage recorded has a high signal-to-electrical noise ratio of 47.3 even when the thin-film is vibrating at small amplitude. The voltage signal is logged for a period of 60 s to ensure the recording contains large enough samples to achieve stochastic convergence despite the random and unpredictable characteristic of turbulent flow. The lateral fluctuations of the thin-film are simultaneously captured for 10 s using an industrial high-speed camera (fps4000-720, UK) at 10^3^ fps. The recorded images of pixel resolution 1280 $\times$ 720 are stored in the camera frame buffer memory before being transferred to the PC for processing. To ensure synchronized measurement of voltage signal and piezoelectric undulation, a simple light-emitting diode (LED) circuit as illustrated in Fig. 1b is employed. Where the start of both recordings is marked by a sharp increase in voltage across the LED just as the LED lights up the moment when circuit is closed. For each set of the experiment, six repeated recordings are performed.

**Table 2:** Dimensions and properties of piezoelectric thin-film^8,16,22^. Subscript *p* represents PVDF film element and subscript *s* the Mylar substrate.

Parameter	Symbol	Value
Length (mm)	*l*	32 ^[m]^
	*l*_*p*_	30
Width (mm)	*b*	16 ^[m]^
	*b*_*p*_	12
Thickness (mm)	*h*	0.22 ^[m]^
	*h*_*p*_	0.028
Young’s modulus (N/m^2^)	*E*_*s*_	3.79 × 10^9^
	*E*_*p*_	3 × 10^9^
Density (kg/m^3^)	*ρ*_*s*_	1390
	*ρ*_*p*_	1780
Second moment of area (mm^4^)	*I*	0.0157 ^[c]^
Strain constant (C/N)	*d*_*31*_	23 × 10^–12^
	*d*_*33*_	− 33 × 10^–12^
Stress constant (Vm/N)	*g*_*31*_	216 × 10^–3^
	*g*_*33*_	− 330 × 10^–3^
Electromechanical coupling factor	*k*_*31*_	12%
	*k*_*t*_	14%
Permittivity (F/m)	*ε*	1.095 × 10^–10^
Capacitance (nF)	*C*	1.38

Superscript [c] indicates calculated and [m] experimentally measured values.

The first series of wind tunnel experiments is conducted to examine the direct fluid–structure interaction of insert-induced turbulent flow and piezoelectric thin-film on the centerline of SFG and RG along different grid-film distances: 5 ≤ *x* ≤ 400 mm i.e. 0.03 ≤ *x*/*D*_*h*_ ≤ 2.50, where *D*_*h*_ = 160 mm is the hydraulic diameter of the wind tunnel. Following that, a preliminary study is performed in the turbulence generation region at *x*/*D*_*h*_ = 0.43 to confirm the symmetrical behaviour of the flow field leeward of SFG on positions P_2_, P_2a_ and P_13_, P_13a_ (see Fig. 2c). In the second series of experiments, the thin-film is positioned in the lower quarter region of SFG and RG (i.e. − 68.5 ≤ *y* ≤ 0 mm and − 67 ≤ *z* ≤ 0 mm corresponding to − 0.43 ≤ *y*/*T* ≤ 0 and − 0.42 ≤ *z*/*T* ≤ 0, respectively) at *x*/*D*_*h*_ = 0.43 as indicated in Fig. 2c,d. It is important to note that all the piezoelectric placements are outside the turbulent boundary layers created by the walls of test section, of which, the boundary layer displacement thickness is estimated to be 3.6 mm at *x*/*D*_*h*_ = 0.43 based on the standard turbulent boundary layer thickness equation in literature^23^. This is to ensure the thin-film is completely vibrating in the grid-induced flow dynamic throughout the entire experiment. Lastly, the thin-film is positioned in the lee of SFG on P_5_ where turbulence forcing is the maximum, with *x*/*D*_*h*_ varying from 0.25 to 0.81 to compare its *x*_*peak*_ against the one obtained on the centerline (P_0_). For each of the positions investigated, the local mean velocities in *x*, *y* and *z*-directions are measured using a hotwire anemometer (Testo 405i, DE) of accuracy ± 0.3 m/s.

### Image-detection algorithm

An in-house thin-film fluctuating image-detection algorithm is developed using MATLAB (version 9.7, USA) to identify the turbulence-induced piezoelectric thin-film tip deflection *δ*. During data post-processing, the camera recorded RGB images are first converted into binary series. Image pixels with luminance greater than certain threshold level are replaced with white pixels, while black pixels are used to substitute the remaining. Noise present in the output images is removed through Area Opening Operation. In addition, boundaries of the thin-film are computed and plotted followed by polynomial curve fitting of the film’s immediate edge to justify the pixel length. Once the ratio of the thin-film physical length scale to pixel dimension, i.e. *r* has been secured, the *δ* can then be determined using,

2 $$\delta = \left( {\frac{{\sum Y_{ins} }}{n} - Y_{ins} } \right) \times r$$

where *Y*_*ins*_ is the instantaneous tip pixel coordinate and *n* is the total number of images processed. To ensure accurate *δ* detection, the luminance threshold during RGB to binary image conversion is set based on the following conditions. (1) Before the fan is turned on, the coordinate of the film at clamped end must match the coordinate obtained via zero-crossing edge-detection algorithm, and (2) the difference between the thin-film’s pixel length for film fluctuation and initial film position are to be less than ± 1%. As a consequence of the current camera resolution and lens limitation, the *δ* detected has an average uncertainty of 0.02 mm.

### Expression of turbulence forcing as a function of thin-film flapping

**Figure 4:**
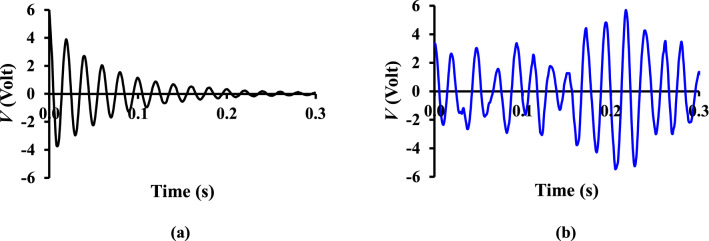
Time history of the piezoelectric voltage *V* (**a**) by lightly flicking the thin-film’s tip in the absence of air flow, and (**b**) at *x*/*D*_*h*_ = 0.425 leeward of SFG centerline.

The electromechanical equation describing the *δ* and voltage *V* generated by a single degree-of-freedom (SDOF) piezoelectric thin-film cantilever beam subjected to an arbitrary force *F* (see Fig. 3) has been previously derived by^24,25^. With the assumptions that the shear forces acting on the beam are very small and that the normal pressure forces are the predominant load^10,11^, the time-dependent normal flow excitation force acting on the piezoelectric thin-film *F*_*y*_ can be expressed as,

3 $$m\ddot{\delta } + c\dot{\delta } + k\delta - \vartheta V = F_{y}$$

where *m*, *c* and *k* are respectively, the mass, damping and stiffness terms for a vibrating piezoelectric beam, while $\vartheta$ denotes the electromechanical coupling coefficient. For a forced vibration of a SDOF mass-spring-damper system, *k* and *c* are defined as follow,

4 $$k = \left( {2\pi f_{n} } \right)^{2} m$$

5 $$c = 2\zeta \sqrt {km}$$

where *f*_*n*_ is the natural frequency and *ζ* the damping ratio. *f*_*n*_ of the piezoelectric thin-film is experimentally determined by lightly flicking the film tip to obtain the dominant frequency of the measured decaying voltage signal (see Fig. 4a) through Fast Fourier Transform (FFT). *ζ* on the other hand can be estimated from the FFT of experimentally detected *δ* using the half-quadratic gain method^26^, namely,

6 $$\zeta = \sqrt {\frac{1}{2} - \sqrt {\left( {4 + 4\left( {\frac{{f_{u} - f_{l} }}{{f_{p} }}} \right)^{2} - \left( {\frac{{f_{u} - f_{l} }}{{f_{p} }}} \right)^{4} } \right)^{ - 1} } }$$

where *f*_*p*_ is the frequency of peak amplitude in the FFT spectrum. *f*_*u*_ and *f*_*l*_ are respectively the frequencies obtained at half-quadratic gain level above and below *f*_*p*_, whereby the amplitudes at half-quadratic gain level are 1/$\sqrt 2$ of the peak amplitude.

It is crucial to mention that the maximum *δ* recorded for the present piezoelectric thin-film is only 20% of its film length, and that the film length is 145 × larger than the film thickness. Based on the classical Euler–Bernoulli beam theory previously used by^14,24,25,27^ to develop the piezoelectric energy harvester models, the deflection of a cantilevered beam undergoing transverse vibration in *y*-direction *y*(*x, t*) can be related to the bending moment *M*(*x, t*) as^28^,

7 $$M\left( {x,t} \right) = EI\left( x \right)\frac{{\partial^{2} y\left( {x,t} \right)}}{{\partial x^{2} }}$$

where *x* is the position along the beam length *L*. For a clamped-free beam with first mode vibration, the mode shape *ϕ*(*x*) at *x* = *L* is defined as^28^,

8 $$\emptyset \left( L \right) = cosh\beta_{1} L - cos\beta_{1} L - \sigma_{1} \left[ {sinh\beta_{1} L - sin\beta_{1} L} \right]$$

The weighted frequency *β*_*1*_*L* = 1.875^28^ while the mode shape coefficients *σ*_*1*_ is given by,

9 $$\sigma_{1} = \frac{{sinh\beta_{1} L - sin\beta_{1} L}}{{cosh\beta_{1} L + cos\beta_{1} L}} = 0.7341$$

From Elvin and Elvin^29^, the $\vartheta$ in Eq. (3) can be deduced as,

10 $$\vartheta = d_{31} E_{p} b_{p} \left( {2h_{a} - h_{p} } \right)\sigma_{1} \beta_{1}$$

where *h*_*a*_ denotes the distance from the top of the PVDF layer to the neutral axis of composite beam. The calculated SDOF piezoelectric beam parameters are tabulated in Table 3. Once *F*_*y*_ in Eq. (3) are secured, the local turbulence forcing of grid-induced flow at various lateral positions and distances streamwise from the grids can be effectively unravelled.

**Table 3:** SDOF piezoelectric beam parameters.

Parameter	Symbol	Value
Mass (g)	*m*	0.17 ^[m]^
Stiffness (N/m)	*k*	17.31
Damping (10^–3^ Ns/m)	c	1.30–7.77
Electromechanical coupling coefficient (10^–6^ C/m)	$\vartheta$	7.69
Natural frequency (Hz)	*f*_*n*_	50.78 ^[m]^
Damping ratio (10^–2^)	*ζ*	1.20–7.16

Superscript [m] denotes experimentally measured values.

### Expression of local equivalent fluid flow undulation via PTFV

The *V* generated by the piezoelectric thin-film in the absence and presence of air flow are plotted in Fig. 4a,b, respectively to demonstrate the role of flow fluctuations in piezoelectric beam vibration response. With a light flick of the piezoelectric thin-film’s tip in the absence of air flow, the *V* amplitude in Fig. 4a oscillates about the equilibrium and decays exponentially to zero in merely 0.3 s. This denotes that the thin-film is undergoing an underdamped vibration as a consequence of mechanical damping. Conversely, when air flow is present, the vortical structures shed from the grid bars induce piezoelectric thin-film vibration to generate a continuous *V* as a function of fluid fluctuating properties (see Fig. 4b). Since piezoelectric transducer is an AC-coupled device where electrical power is virtually generated from the turbulence fluctuations in the flow, and that the average power harvested exhibits trend and behaviour that is similar to that of the turbulence kinetic energy^11^, we could therefore infer that *V* and *δ* are interrelated with the local flow undulation. Hence in the present study, we calculate the local equivalent lateral flow velocity fluctuation *v′* from the time derivative of experimentally detected *δ*, i.e., $\frac{d\delta }{{dt}}$. We stress that the *v′* calculated represents the velocity fluctuation that is qualitatively, but not quantitatively equivalent to the actual fluid fluctuating velocity obtained using direct hot-wire measurements. The various equivalent turbulence statistics deduced from the present *v′ *will be discussed in the following section to provide insights into the mechanical characteristics of grid-induced turbulent flow.

## Results and discussion

### Effects of grid-film distance on insert-induced flow characteristics

**Figure 5:**
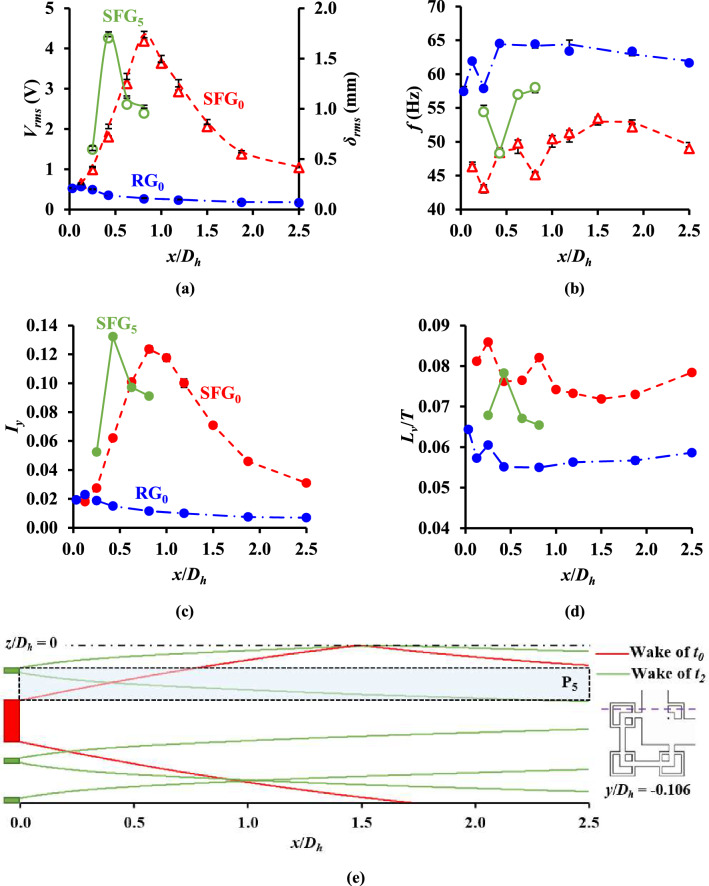
(**a**) RMS of experimentally recorded piezoelectric output voltage *V*_*rms*_ and tip deflection *δ*_*rms*_, as well as (**b**) the corresponding dominant frequency of voltage signal *f*_*V*_ and thin-film flapping *f*_*δ*_, at different streamwise distances *x*/*D*_*h*_ along the leeward of SFG(*P*_0_, *P*_5_) and RG centerline [*note*: Symbol denotes piezoelectric measurements and line the camera detection]. Grid-induced equivalent (**c**) turbulence intensity *I*_*y*_ and (**d**) lateral integral length scale *L*_*v*_*/T* against *x*/*D*_*h*_. (**e**) Interactions between wakes generated by the SFG bars at *y*/*D*_*h*_ = − 0.106.

Figure 5a presents the RMS voltage *V*_*rms*_ and RMS tip deflection *δ*_*rms*_ of the piezoelectric thin-film recorded at various streamwise distances *x*/*D*_*h*_ along SFG(P_0_, P_5_) and RG centerline, with their corresponding dominant frequencies *f*_*V*_ and *f*_*δ*_ shown in Fig. 5b. The local equivalent turbulence intensity *I*_*y*_ in *y*-direction, defined as the ratio of RMS *v′* to the inlet velocity (*v′*_*rms*_/*v*_*in*_) is plotted against *x*/*D*_*h*_ in Fig. 5c. The autocorrelation function of *v′* is obtained to determine its internal correlation within a time series by comparing *v′* at time *t* with that at time lag *k*. The first zero-crossing *τ*_*zc*_ of the autocorrelation plot denotes the period at which *v′* decorrelates from the primarily induced turbulence. By integrating the autocorrelation function of *v′*, the size of the large energy-containing eddies, viz. the equivalent lateral integral length scale *L*_*v*_ is calculated as per Eq. (11). A comparison between the *L*_*v*_ for SFG_0,_ SFG_5_ and RG_0_ are depicted in Fig. 5d, where *L*_*v*_ is normalized against the tunnel width *T*.

11 $$L_{v} = v_{in} \mathop \smallint \limits_{0}^{{\tau_{zc} }} \frac{{\langle v^{\prime}_{t} v^{\prime}_{t + k} \rangle}}{{\langle v^{{\prime}{2}}\rangle }}dk$$

Figure 5a,c reveal the existence of two turbulent regions, viz. the turbulence generation and decay regions as described in literature^7,21,30^. The *V*_*rms*_, *δ*_*rms*_ and *I*_*y*_ induced by SFG-generated turbulence along P_0_ and P_5_ increase, respectively in the generation region to a peak at *x*_*peak*_/*D*_*h*_ = 0.813 and 0.425, which are then decrease exponentially further downstream in the decay region. For RG, the values reach a peak at *x*_*peak*_/*D*_*h*_ = 0.125 which are 5 to 7 × lower than SFG_0_ before experiencing a power-law decay with respect to *x*/*D*_*h*_. The overall 5 × higher *I*_*y*_ flow generated by the SFG along the centerline as compared to RG of similar *σ* is in line with the previous studies^7,20^. Essentially, the vorticity of vortical structures (eddies) shed from the grid bars induces fluctuations in velocity perpendicular to the piezoelectric thin-film surface which brings fluid up and down towards the thin-film^11^. Hence, higher *I*_*y*_ promotes higher conversion of induced fluid dynamic perturbation into vibrational energy, giving rise to a 6 × higher average *V*_*rms*_ and *δ*_*rms*_ attained downstream of SFG_0_ in comparison to RG_0_. It is also important to note that although the signal-to-noise ratio of *δ* for small thin-film undulation is 9.3 × lower than the voltage signal-to-electrical noise ratio, the primary signal, or the large-scale fluctuations of the *δ* and *V* responses are rather well fitted to each other as depicted in Fig. 12b. Therefore, the statistical results deduced from the camera recorded *δ* are deemed reliable as evidenced in Fig. 5a,c where similar profiles are obtained for *V*_*rms*_, *δ*_*rms*_ and *I*_*y*_.

The further peak point observed for SFG_0_ as compared to SFG_5_ in Fig. 5a,c is due to the wake interactions of different-size bars at different *x*/*D*_*h*_. The typical wake width *w* at a streamwise distance *x* based on a wake-generating bar of thickness *t* is approximated as $w\sim\sqrt {xt}$ ^21^. Figure 5e illustrates the possible wake interactions originating from the bars of SFG at *y*/*D*_*h*_ = − 0.106. Along position P_5_ (shaded in blue), the wakes generated by the largest bar and smallest bar nearest to the midplane interact at a distance *x*/*D*_*h*_≈0.4. This location is considerably closer to the grid as compared to the interaction between the wakes of largest grid bars on the centerline which occurs at $x_{*}$/*D*_*h*_ = 1.5. On the contrary, the peak location for RG_0_ occurs closest to the grid since RG has a smaller $x_{*}$ compared to SFG. In the present study, *x*_*peak*_/$x_{*}$ = 0.54, 0.28 and 0.29 for SFG_0_, SFG_5_ and RG_0_, respectively. The larger *x*_*peak*_/$x_{*}$ value attained for SFG_0_ as compared to the 0.45 constant determined by Mazellier and Vassilicos^21^ is due to our current SFG having a *σ* which is almost double than that of their grids. Such high *σ* is a direct result of the large *t*_*0*_/*L*_*0*_ ratio (0.32) which enlarges the size of the recirculation regions immediately leeward the largest grid bars, causing the streamlines of the flow to diverge towards the tunnel walls^7^. This leads to a delay in the wake-interaction from the largest bars and prompts the wakes to meet further downstream.

As seen in Fig. 5b, although *f*_*V*_ and *f*_*δ*_ in the lee of SFG have variations of less than 15% from the *f*_*n*_ of the piezoelectric beam, its vibration frequency is affected to some extent, by the characteristic frequency of the vortices shed from the grid bars. The overall dominant frequencies *f* of SFG_0_ are the lowest, followed by SFG_5_ and lastly RG_0_. This can be explained by the larger overall *L*_*v*_/*T* of SFG_0_ evident in Fig. 5d, of which large, slowly rotating eddies possess lower frequency while small eddies experience rapid fluctuations. As a result, the *L*_*v*_/*T* profiles are a complete reverse of the *f* profiles. For the case of SFG_0_, the *L*_*v*_/*T* calculated are the same order of magnitude as those determined by Hurst and Vassilicos^30^. The *L*_*v*_ for SFG_0_ ranges from 11.5 to 13.7 mm, which on average is 1.1 to 1.3 × larger than SFG_5_ and RG_0_. In grid-generated turbulence, the size of the largest eddies is set by the characteristic geometric length scale of the mean flow. Since *L*_*0*_ is smaller for RG, its *L*_*v*_/*T* are considerably lower than SFG, whereas the difference in *L*_*v*_/*T* for SFG_0_ and SFG_5_ are due to the multi-scale vortices shed from the multiple-length bars of SFG.

From Fig. 5b,d, we further verify the existence of two distinct flow dynamic regimes downstream of the SFG. The highly irregular *f* and *L*_*v*_/*T* observed near the grid corresponds to the turbulence generation region where the flow is inhomogeneous and anisotropic, while the relative uniform *f* and *L*_*v*_/*T* obtained further downstream signifies the homogeneous and isotropic flow in the decay region. Additionally, it is observed that there is a drop in *f* when *V*_*rms*_ and *δ*_*rms*_ are the maximum for SFG_0_ and SFG_5_. Low *f* reflects large-scale energy-containing turbulent eddies with significant amplitude of velocity fluctuations, acting as the primary components of the driving mechanism for thin-film flapping. Hence, a peak in film bending and voltage generation is generally accompanied by an increase in *L*_*v*_/*T* and a drop in *f*. This behaviour however, is not observable for the case of RG. Interestingly, even though the eddies for SFG_5_ at *x*_*peak*_/*D*_*h*_ = 0.425 have a smaller *L*_*v*_/*T* and higher *f* compared to SFG_0_ at *x*_*peak*_/*D*_*h*_ = 0.813, their *V*_*rms*_ and *δ*_*rms*_ are almost equivalent, with the *I*_*y*_ of SFG_5_ to be 1.1 × higher than SFG_0_. The wake-interaction phenomenon illustrated in Fig. 5e could provide a possible explanation, of which, *x*_*peak*_ of SFG_5_ is in close proximity to the location where the wake from the largest bar encounters the wake from the smallest bar nearest to the midplane. This implies that wake interactions could further intensify the local turbulence strength generated by the SFG which adds to the higher-than-expected peak responses obtained for SFG_5_. Conversely, whilst the peak point on the centerline is bounded by the location where the inward spreading wakes from the largest bars interact with each other, its peaks are mainly attributable to the higher energy containing large-scale effective flow structures shed from the grid bars.

**Figure 6:**
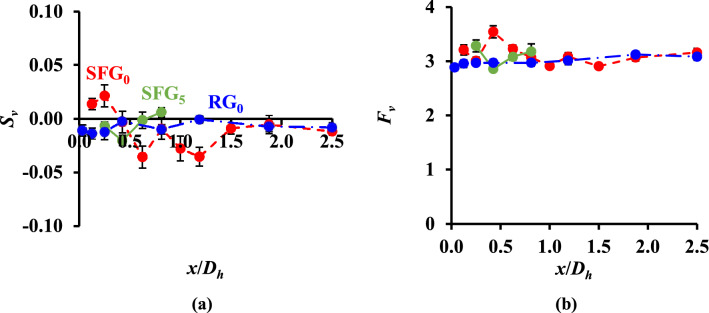
(**a**) Thin-film undulation skewness *S*_*v*_, and (**b**) flapping flatness *F*_*v*_, against *x*/*D*_*h*_.

To comprehend the Gaussianity of *v′*, we observe the skewness *S*_*v*_ (Fig. 6a) and flatness *F*_*v*_ (Fig. 6b) of *v′* against *x*/*D*_*h*_ for SFG_0_, SFG_5_ and RG_0_, where *S*_*v*_ and *F*_*v*_ are computed as,

12 $$S_{v} = \frac{{\left\langle {v^{{{\prime }3}} } \right\rangle }}{{\left\langle {v^{{{\prime }2}} } \right\rangle ^{{3/2}} }}$$

13 $$F_{v} = \frac{{\left\langle {v^{{\prime 4}} } \right\rangle }}{{\left\langle {v^{{\prime 2}} } \right\rangle ^{2} }}$$

As shown in Fig. 6a,b, both grids reveal *S*_*v*_ and *F*_*v*_ values correspond to Gaussian distribution (*S*_*v*_≈0, *F*_*v*_≈3) except for SFG_0_ at *x*/*D*_*h*_ = 0.43, where *F*_*v*_ has a relatively larger magnitude (*F*_*v*_ = 3.5), suggesting that the *v′* are generally close to zero yet prone to extreme fluctuations sporadically. Furthermore, SFG demonstrates higher variations in *S*_*v*_ and *F*_*v*_ compared to the rather uniform values obtained for RG at all *x*/*D*_*h*_ as a consequence of free decay homogenous isotropic turbulence^31^. The variability of *S*_*v*_ and *F*_*v*_ with *x*/*D*_*h*_ could be an indication of the inhomogeneity in fractal grid-generated turbulence particularly in the turbulence generation region. Nevertheless, the extraordinary large longitudinal velocity flatness *F*_*u*_ and negative velocity skewness *S*_*u*_ observed by^21,32^ along the centerline at *x*/$x_{*}$≈0.2 are not evident in Fig. 6a,b. We postulate that these intense decelerating flow events either do not occur for a lateral flow or, the inherited strength is rather inferior. This could be due to the complex interaction between the flow and the thin-film structure as compared to direct hot-wire measurements, in which PTFV may have potentially filtered out information relating to the higher order statistics of turbulent flow. However, more research is needed to confirm this hypothesis, such as the use of different geometrical and mechanical properties’ piezoelectric thin-film to examine how the inherited strength may be responsible for the strong suppression of the non-Gaussian behaviour of *v′*.

**Figure 7:**
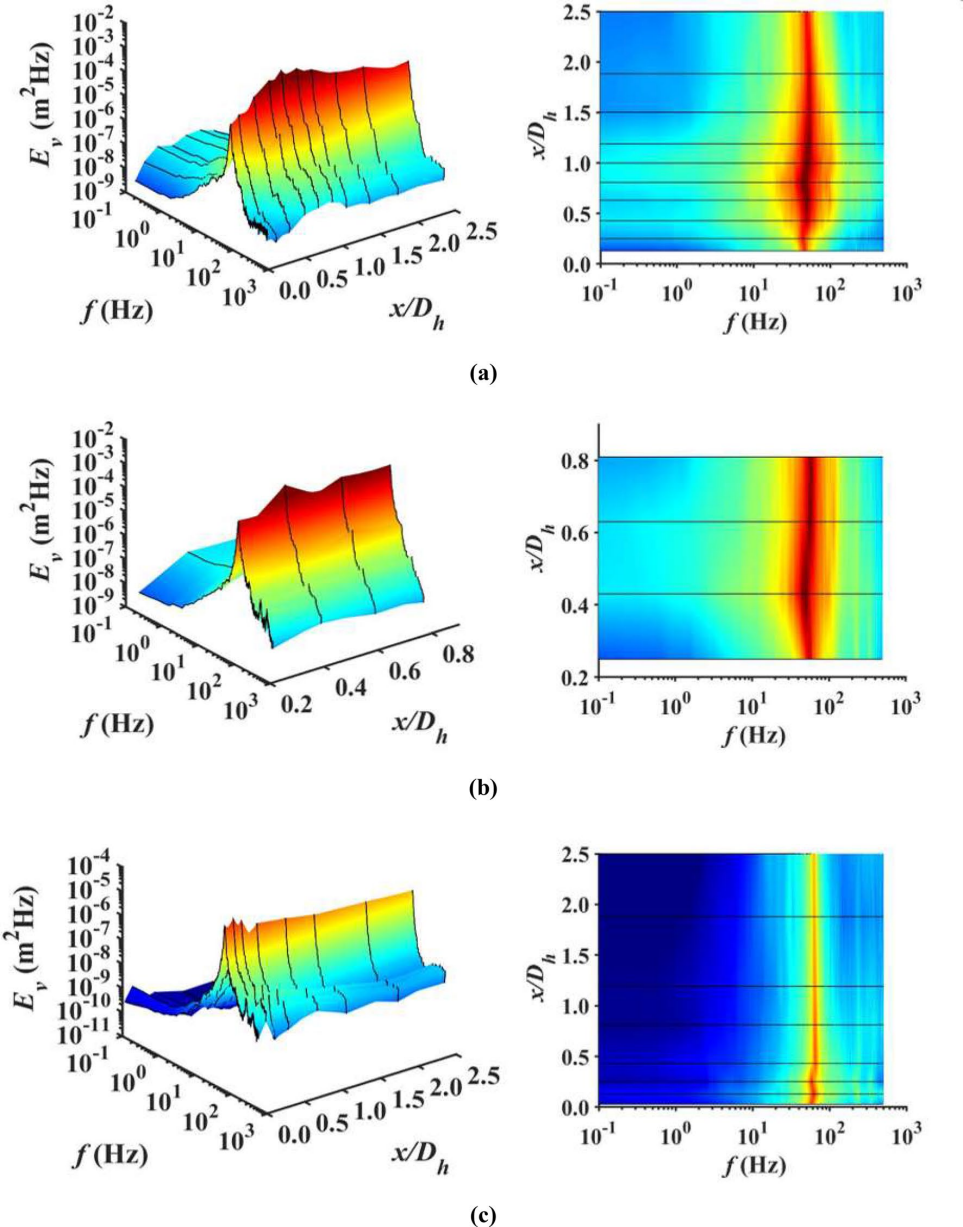
Downstream evolution of the energy spectrum (left) on (**a**) *P*_0_, (**b**) *P*_5_ of SFG, and (**c**) centerline of RG; top view of the respective spectrum (right).

Figure 7a–c present the streamwise energy spectra *E*_*v*_ for SFG_0_, SFG_5_ and RG_0_ with the left column of figures showing the 3D downstream flow dynamic’s energy spectra, and top view along the right. The energy spectra are obtained from the Fourier transform of the autocovariance function, i.e., *v′*. As shown in Fig. 7, there appears to be multiple peaks in the spectrum for individual streamwise distance, indicating that energy is distributed over a multitude of vortices with a wide range of spatial scales and characteristic frequencies. The major peaks occurring near frequency *f*≈49 Hz, 55 Hz and 62 Hz, respectively for SFG_0_, SFG_5_ and RG_0_ are the results of vortex shedding from the grid bars^33–35^. One can infer that the vortex shedding effect from the SFG bars is more pronounced, with the major peak *E*_*v*_ at *x*_*peak*_/*D*_*h*_ leeward of SFG to be approximately 30 × higher in comparison to RG. Such intense vortex shedding observed for the case of SFG_0_ could also have masked the non-Gaussian behaviour of *v′* in the turbulence generation region^33^, causing the corresponding *S*_*v*_ and *F*_*v*_ values in Fig. 6a,b to be close to 0 and 3, respectively. Moreover, more energetic fluctuations (*E*_*v*_ > 10^–5^) are detected for a broader range of *f* in the turbulence generation region leeward of SFG_0_ (22 Hz < *f* < 98 Hz) and SFG_5_ (29 Hz < *f* < 85 Hz) as compared to RG_0_ (53 Hz < *f* < 69 Hz), suggesting that SFG-induced turbulence contains more effective flow structures contributing to the thin-film undulation.

The *f* corresponding to the major peaks for all three cases are in excellent agreement with the *f*_*V*_ and *f*_*δ*_ determined earlier in Fig. 5b, of which, they are associated to the first resonant mode of piezoelectric beam amalgamate with their respective vortex shedding frequencies. In addition, the streamwise evolution of the maximum *E*_*v*_ are consistent with the trend previously observed in Fig. 5a,c. One can therefore deduce that more energetic vortex shedding gives rise to higher turbulence intensity flow that yields larger film bending and consequently higher voltage output. On the other hand, minor peaks with relatively smaller *E*_*v*_ tend to appear at higher *f* close to the second vibration mode of the piezoelectric beam. These peaks are more marked in RG in comparison with SFG, especially in the domain proximate to grid, denoting that higher frequency small-scale eddies possess more significant contribution to RG-induced turbulence. As one proceeds downstream in the decay region, both the mechanically deduced major and minor peaks diminish due to viscous dissipation, with the minor peak intensity of RG attenuating up to 3× faster than its major peak, since smaller eddies of higher characteristic frequencies decay faster.

### Streamwise evolution of grid-induced turbulence forcing

**Figure 8:**
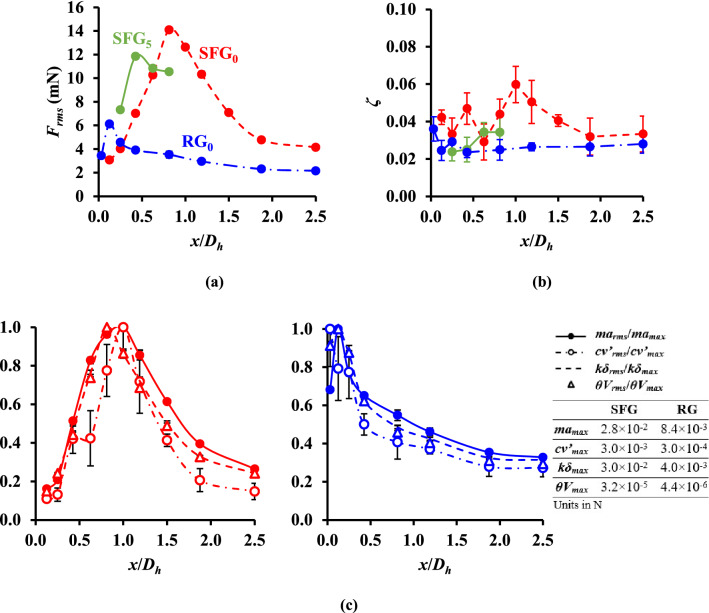
Grid-induced (**a**) RMS excitation force acting on the piezoelectric thin-film *F*_*rms*_ and (**b**) damping ratio *ζ* against *x*/*D*_*h*_. (**c**) Downstream profile of the normalized acceleration *ma*_*rms*_/*ma*_*max*_, velocity *cv′*_*rms*_/*cv′*_*max*_, deflection *kδ*_*rms*_/*kδ*_*max*_ and voltage *θV*_*rms*_/*θV*_*max*_ terms along the centerline of SFG (red) and RG (blue).

To demonstrate the turbulence forcing of grid-induced flow at various distances leeward from SFG_0_, SFG_5_ and RG_0_, the RMS of the normal flow excitation force *F*_*rms*_ acting on the thin-film was computed as per Eq. (3) and plotted in Fig. 8a. The *F*_*rms*_ calculated in the present study are in the range of 2 to 14 mN, which are consonant with the millinewton excitation force dynamically measured by Goushcha et al.^10^ in their turbulent boundary layer study with momentum thickness-based Reynolds number: 2.0 × 10^3^ ≤ *Re*_*θ*_ ≤ 7.5 × 10^3^.

As seen in Fig. 8a, the *F*_*rms*_ for all three cases display a similar streamwise flow dynamic evolution as the results in Fig. 5a,c. The turbulence generated by SFG_0_ generally possesses larger forcing compared to RG_0_ except for *x*/*D*_*h*_ ≤ 0.25. At *x*_*peak*_, the turbulence forcing of fractal grid-induced flow on the centerline is more than twice of that induced by RG. Perhaps more interestingly may be the observation that the *F*_*rms*_ for SFG_5_ has a lower peak than SFG_0_ despite both having almost the same peak *V*_*rms*_ and *δ*_*rms*_ (see Fig. 5a). This could be explained by the higher equivalent vortex shedding energy of SFG_0_ in comparison to SFG_5_ as represented by the wider *f* range of high energy fluctuations in Fig. 7. The same explanation applies to the case of RG_0_ and SFG_0_ at *x*/*D*_*h*_ = 0.13, where the former has an equivalent vortex shedding energy distributed among a range of frequencies that is 1.3 × broader than the latter. Moreover, the small-scale eddies of RG_0_ possess more energy in exciting the thin-film considering that the *E*_*v*_ of its minor peak is an order of magnitude larger than SFG_0_. From these findings, we can deduce that the experimentally recorded *V*_*rms*_ and *δ*_*rms*_ reflect only the vortex shedding intensity of dominant large eddies. Conversely, the calculated *F*_*rms*_ takes into consideration the broad-band random forcing across various eddy sizes including small-scale eddies of larger characteristic frequencies.

Figure 8b shows the damping ratio *ζ* of the thin-film vibration computed from Eq. (6). The film oscillations for all three cases are underdamped, with *ζ* < 0.1. From the *ζ* evolution seen in Fig. 8b, the average *ζ* for SFG_0_ is 1.5 × higher than RG_0_, signifying a higher thin-film damping in fractal grid-generated turbulence. This might be due to the complicated multilength-scale flow structures of SFG that flow through the thin-film over time, making the fluid domain around the thin-film becomes rather “crowded” yet highly mechanical effective.

The forced vibration of piezoelectric thin-film is the result of the interplays between fluid excitation force, inertia force, viscous effect and oscillating piezoelectric structure. Here we normalize the RMS of acceleration *ma*_*rms*_, velocity *cv′*_*rms*_, deflection *kδ*_*rms*_ and voltage *θV*_*rms*_ terms in Eq. (3) by their respective maximum over the streamwise distances. The downstream profile of the normalized terms (*ma*_*rms*_/*ma*_*max*_, *cv′*_*rms*_/*cv′*_*max*_, *kδ*_*rms*_/*kδ*_*max*_ and *θV*_*rms*_/*θV*_*max*_) along SFG_0_ and RG_0_ are depicted in Fig. 8c with their respective maximum terms listed in the side table. Clearly, the downstream evolution of all the four normalized terms for both grids are almost similar to the *F*_*rms*_ profiles in Fig. 8a with the exception of the velocity term. Where *cv′*_*rms*_/*cv′*_*max*_ peaks at *x*/*D*_*h*_ = 1.0 for SFG_0_ and *x*/*D*_*h*_ = 0.03 for RG_0_ considering that the *ζ* at these two locations are the highest, respectively. One can also see that all the four terms are an order of magnitude higher for SFG_0_ when compared against RG_0_.

From the table in Fig. 8c, one can infer that the force acting on the piezoelectric cantilever beam is generally dominated by the inertia (*ma*) and bending forces (*kδ*) followed by the mechanical damping force (*cv′*) of an order of magnitude smaller. The momentum exchange between turbulent fluctuations generated from the grids contributes predominantly towards the beam forcing in the form of inertia force. The splashing of high momentum fluid transported by the moving vortical structures on the thin-film surface creates a considerable amount of bending force that causes the beam to deflect. At the same time, energy is lost through mechanical damping, viz. viscous air damping and structural damping. The dissipation of the electrical charges generated by piezoelectric through the circuit will also act to dampen out the beam vibration. In view of the low viscosity of air and high elastic compliance of current piezoelectric thin-film employed, mechanical damping force has a relatively minor effect on the beam forcing. Hence, SFG has an overall greater *F*_*rms*_ than RG in spite of the higher thin-film damping imposed by fractal grid-generated turbulence. On the other hand, the force due to electromechanical coupling (*θV*) has an order of magnitude between 10^–6^ and 10^–5^ which is almost negligible, signifying that the electrical damping imposed by the present PVDF film is insignificant.

### Cross-sectional turbulence mechanical characteristics in the lee of SFG and RG

**Figure 9:**
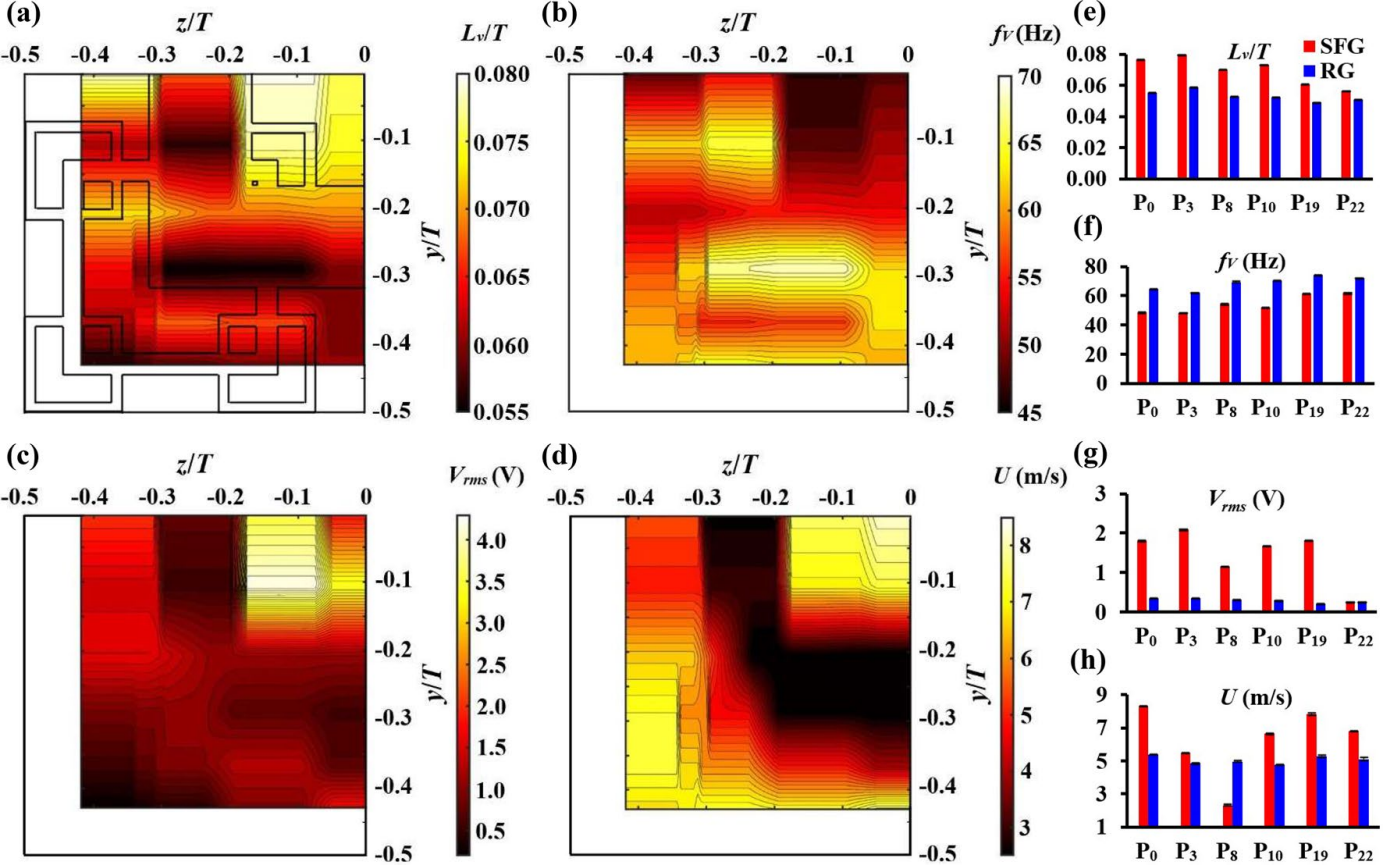
2D contours of (**a**) *L*_*v*_/*T*, (**b**) *f*_*V*_, (**c**) *V*_*rms*_ and (**d**) local mean velocity *U* at *x*/*D*_*h*_ = 0.425 leeward of SFG, and (**e**–**h**) the corresponding results for RG at positions *P*_0_, *P*_3_, *P*_8_, *P*_10_, *P*_19_ and *P*_22_.

In order to have a better comprehension of the grid’s cross-sectional turbulence mechanical characteristics in the turbulence generation region, the *V* and *δ* of piezoelectric thin-film at *x*/*D*_*h*_ = 0.425 downstream of SFG and RG were recorded by placing the thin-film at the lateral positions indicated in Fig. 2c,d. Table 4 compares the results obtained for SFG(P_2_, P_2a_) and SFG(P_13_, P_13a_). All their results are seen to be respectively close to each other, which proves that the flow field leeward of SFG are symmetry and therefore, the present study focuses mainly on the lower quarter grid. Figure 9a–d illustrate the 2D contours of *L*_*v*_/*T*, *f*_*V*_, *V*_*rms*_ and local mean velocity *U* for SFG, of which, *U* is defined as,

14 $$U = \sqrt {U_{x}^{2} + U_{y}^{2} + U_{z}^{2} }$$

where *U*_*x*_, *U*_*y*_ and *U*_*z*_ are respectively the local mean velocities in *x*, *y* and *z*-directions measured using hotwire anemometer. The corresponding results obtained for RG at positions P_0_, P_3_, P_8_, P_10_, P_19_ and P_22_ are plotted against SFG in Fig. 9e–h. Note that *δ*_*rms*_ and *I*_*y*_ are omitted owing to the similarity with respect to *V*_*rms*_ in Fig. 9c,g.

**Table 4:** Results obtained for SFG at positions P_2_ vs. P_2a_ and P_13_ vs. P_13a_.

Position	*V*_*rms*_ (V)	*f*_*V*_ (Hz)	*F*_*rms*_ (mN)	*ζ*	*L*_*v*_/*T*	*S*_*v*_	*F*_*v*_	*U* (m/s)
P_2_	0.8	54.0	4.9	0.050	0.068	− 0.023	3.5	2.6
P_2a_	0.7	54.3	4.2	0.072	0.069	− 0.021	3.4	3.2
P_13_	1.1	68.5	9.2	0.032	0.055	− 0.003	3.1	4.8
P_13a_	1.1	67.7	9.8	0.034	0.055	− 0.004	3.1	4.4

From Fig. 9a,b, an immediate observation that we can make is the variability of *L*_*v*_/*T* and *f*_*V*_ throughout the entire contour owing to the multi-scale nature of SFG as well as the inhomogeneity of fractal grid-generated flow in the turbulence generation region. The *L*_*v*_ of the vortices shed from the different-size bars of SFG ranges from 8.7 to 12.8 mm with an average cross-sectional *f*_*V*_ of 58 Hz. Furthermore, it is apparent that the cross-sectional profile of *f*_*V*_ is a complete reverse of *L*_*v*_/*T*, which is consistent with our earlier finding. Large-scale, slow rotating eddies are mostly found at the central opening of the SFG, whereas small-scale eddies with high characteristic frequencies are mainly observed in the wake of the largest grid bar as well as at the bottom left corner of the contour. This observation agrees well with the *V*_*rms*_ result in Fig. 9c, of which, large energy-containing eddies induce larger thin-film bending, giving rise to a higher voltage output. On the other hand, energy dissipates faster in smaller size eddies, leading to lower voltage generated. We hypothesize that the small-scale eddies present at bottom left of the contour is the result of vortices shed from smaller size grid bars near the corner of SFG, as well as the breaking up of large-scale eddies into smaller ones when flow strikes the side and bottom walls of test section. This could also explain the slight decrease in *L*_*v*_/*T* and the increase in *f*_*V*_ for both grids as one approaches the bottom of the test section (see Fig. 9e,f). At each of the lateral positions investigated, RG has a smaller *L*_*v*_/*T* but higher *f*_*V*_ compared to SFG.

**Figure 10:**
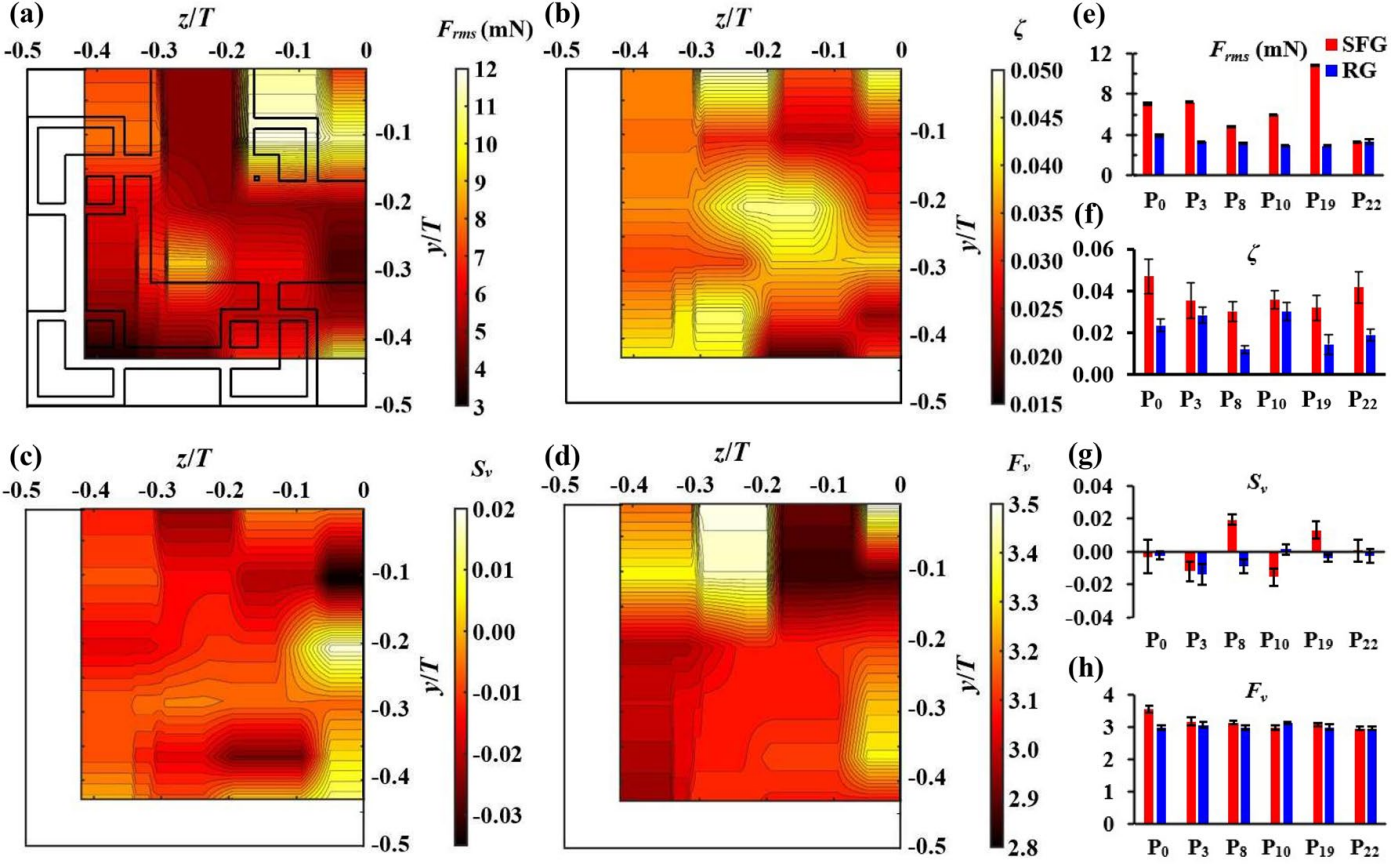
(**a**) *F*_*rms*_, (**b**) *ζ*, (**c**) *S*_*v*_ and (**d**) *F*_*v*_ at different lateral positions of SFG and (**e**–**h**) RG where *x*/*D*_*h*_ = 0.425.

In Fig. 9c, high *V*_*rms*_ is observed in the region between the largest grid bar and centerline of SFG with P_5_ having the greatest *V*_*rms*_. This can be explained by the high velocity jet flowing through the grid opening (see Fig. 9d) as well as the wake-interaction occurring at P_5_ as discussed previously (see Fig. 5e). On the contrary, *V*_*rms*_ is low on the rear side of the largest grid bar due to the presence of recirculating flow as represented by the low velocity wake region in Fig. 9d. Low *V*_*rms*_ is also detected at the bottom left corner of the contour albeit the high *U*, with P_22_ having the lowest *V*_*rms*_. This could be a consequence of the chaotic flow stirred up by the aforementioned small-scale eddies in the bottom left region of the test section. The force acting on the thin-film provided by the highly disordered eddies would most likely counteract each other, imposing thin-film damping upon fractal grid-generated turbulence as supported by the high *ζ* in Fig. 10b. On average, the cross-sectional *V*_*rms*_ in the lee of SFG are 5× higher than the RG in Fig. 9g. At P_8_, SFG has a lower *U* but higher *V*_*rms*_ than RG (see Fig. 9g,h) owing to the 3× higher localized *I*_*y*_ flow generated by SFG behind the largest grid bar.

**Figure 11:**
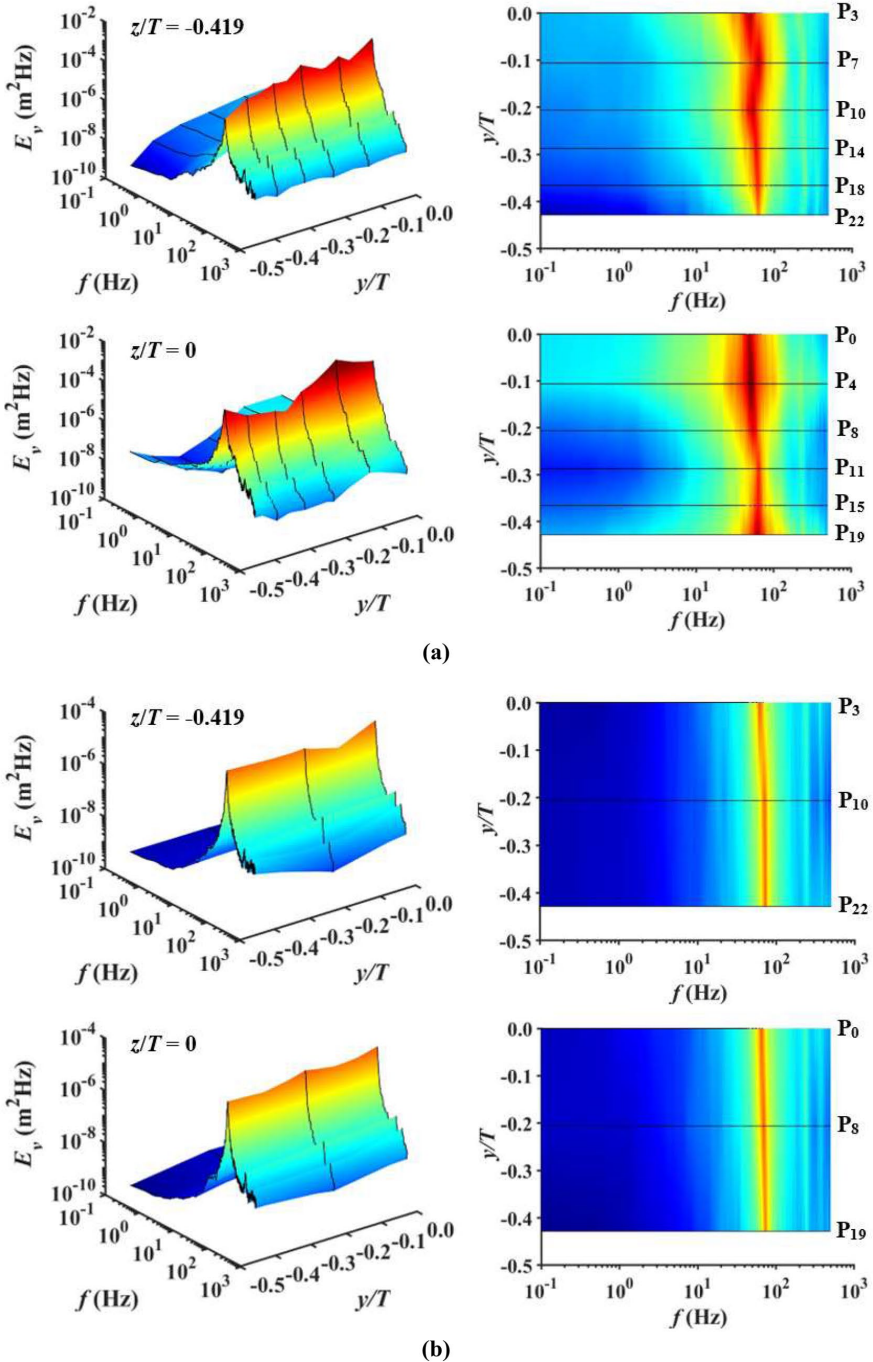
Lateral variations in energy spectrum (left) along *z/T* = − 0.419 and *z/T* = 0 at *x*/*D*_*h*_ = 0.425 leeward of (**a**) SFG and (**b**) RG; top view of the respective spectrum (right).

The 2D contours of *F*_*rms*_, *ζ*, *S*_*v*_ and *F*_*v*_ for SFG are presented in Fig. 10a–d. The results are compared against RG(P_0_, P_3_, P_8_, P_10_, P_19_, P_22_) and plotted in Fig. 10e–h. With the exclusion of SFG(P_12_, P_13,_ P_19_), the *F*_*rms*_ in Fig. 10a generally displays a similar profile as the *V*_*rms*_ in Fig. 9c. By taking P_0_ as the baseline, one can see that both P_0_ and P_19_ have comparable *V*_*rms*_ due to the same intensity of vortex shedding, i.e., same maximum *E*_*v*_ (see Fig. 11a). However, due to the unique geometry of SFG, the small-scale eddies on P_19_ possess higher energy than P_0_ as depicted by the higher *E*_*v*_ of the minor peak near *f* = 240 Hz, giving rise to a larger *F*_*rms*_ upon thin-film flapping. Applying a similar analysis, P_13_ has a lower *V*_*rms*_ than P_0_ due to the lower shedding intensity of faster rotating yet smaller *L*_*v*_ vortices from the second iteration grid bar. Nevertheless, the additional forcing provided by the higher *E*_*v*_ small eddies on P_13_ gives rise to a larger *F*_*rms*_ as compared to P_0_. Moving on to P_12_, the flow recirculation behind the largest grid bar leads to a less pronounced vortex shedding effect but with no significant impact on the energy content of small-scale eddies, thus the discrepancy in the *F*_*rms*_ and *V*_*rms*_ on P_12_. As seen in Fig. 10e, the cross-sectional turbulence forcing of the RG-generated flow is comparatively uniform, which on average is 2× lower than the SFG-induced turbulence. The present findings further justify our speculations made earlier that *F*_*rms*_ expresses the force acting on the thin-film contributed by various scales flow structures, including the additional forcing provided by small-scale turbulence with less significant amplitude of velocity fluctuation.

From Fig. 10b,f, we once again observe that the film oscillations at all the lateral positions for both grids are underdamped, with SFG having an average *ζ* that is 1.5× higher than RG. As discussed previously, the high *ζ* in the bottom left region leeward of SFG is due to the highly chaotic fluid domain at the corner of the test section, whereas the high *ζ* behind the largest grid bar is caused by the recirculating flow in the wake region. On the other hand, the jet-like behaviour through the opening is the strongest on the centerline of SFG. When this strong central jet flows over the film surface, it may possibly hinder the upward and downward movement of thin-film, causing a less effective thin-film flapping as supported by the relatively higher *ζ* on P_0_ than P_1_ and P_4_, thence lower *F*_*rms*_ and *V*_*rms*_.

It is observed in Fig. 10c,d,g,h that the *v′* distributions for both SFG and RG are Gaussian. This is excepted for SFG(P_0_, P_2,_ P_6_), where *F*_*v*_≈3.5, implying that most of the time *v′* are near-zero with extreme fluctuations occurring occasionally when larger size eddies of considerable velocity fluctuation amplitude impinge on the thin-film surface. It is also noteworthy to mention that we do not observe any intense accelerating or decelerating events occurring on all the lateral positions investigated for SFG, in contrast to the inhomogeneous *S*_*u*_ and *F*_*u*_ cross-sectional profiles reported by Nagata et al.^36^ in the turbulence generation region. Given that the displacement of an elastic structure in response to the random turbulence-induced pressures on its surface can be well represented by a Gaussian distribution^37^, it is reasonable to infer that our *S*_*v*_ and *F*_*v*_ reflect only the Gaussianity of the thin-film’s structural response towards turbulent flow. Hence, the present PTFV approach might not be feasible in characterizing the higher order statistics of turbulent velocity fluctuations, instead allows the unique expression of insert-induced turbulence mechanical characteristics.

Figure 11a,b display the lateral evolution of energy spectra along *z*/*T* = − 0.419 and *z*/*T* = 0 at *x*/*D*_*h*_ = 0.425 in the lee of SFG and RG with the 3D contour presented in the left column, and top view on the right. The immediately notable observation for both grids is that the lateral variations of maximum *E*_*v*_ from the middle to the bottom wall of test section are identical to the *V*_*rms*_ trends in Fig. 9c,g. It can also be observed that an increase in the *E*_*v*_ of major peak is generally accompanied by a wider *f* range of high energy fluctuations as shown in the right side of Fig. 11. Hence, more intense vortex shedding gives rise to more effective flow structures responsible for the higher voltage generated through piezoelectric thin-film flapping. The vortex shedding intensity of SFG is found to be 7 to 37× higher than RG with the exception of P_22_ owing to the lower shedding intensity of high frequency, low energy containing vortices from the local higher iteration grid bars. Moreover, the breaking up of vortical structures by the corner walls as previously discussed could also have possibly weaken the vortex shedding effect. The vortex shedding phenomenon is also observed to be less pronounced on SFG(P_8_, P_11_) since they are both located in the wake of the largest grid bar where flow recirculation occurs. As one advances towards the bottom wall, the *f* corresponding to the major peak increases from 48 to 62 Hz for SFG and 61 to 74 Hz for RG, which agrees well with the *f*_*V*_ results in Fig. 9b,f. For all the lateral positions investigated, we once again secure a more prominent minor peak for RG with respect to SFG except for P_22_, signifying that the contribution of small-scale eddies towards *v′* is very significant for SFG near the corner walls.

### A probabilistic description of piezoelectric voltage response towards grid-induced turbulence

**Figure 12:**
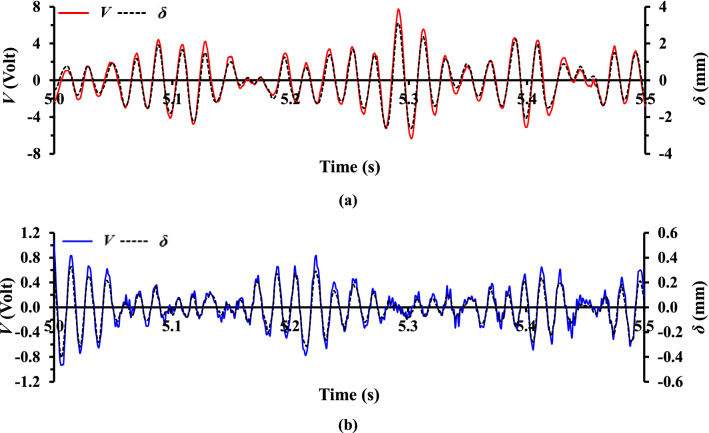
Time series plots of *V* and *δ* recorded at *x*/*D*_*h*_ = 0.425 downstream of (**a**) SFG and (**b**) RG centerlines.

Figure 12a,b show a segment of the recorded time-history for *V* and *δ* at *x*/*D*_*h*_ = 0.425 leeward of SFG and RG centerlines. The intermittent behavior observed for both grids are mainly due to the random nature of grid-induced turbulence impingement on the thin-film, of which, the *V* and *δ* for SFG are about 6× greater than that of RG. Although at first glance the *V* and *δ* responses for both grids seem well fitted, the small-scale fluctuations are not well matched to each other particularly for the RG in Fig. 12b. These small-scale fluctuations are the result of minute eddies impinging on the thin-film which may either intensify or offset the primary film oscillation induced by the large-scale eddies. Nonetheless, if the large eddies contain high enough energy to cause considerable film bending as seen for the case of SFG in Fig. 12a, it will mask the relatively insignificant fluctuations caused by the small eddies. These results are in good agreement with the energy spectra in Fig. 11, where a much prominent minor peak is observed for RG_0_ as compared to SFG_0_. Furthermore, the small-scale fluctuations are mostly visible in the *V* response since the output voltage is based on the overall piezoelectric deformation induced by the multilength-scale eddies along the thin-film surface, instead of merely the flow structures revolving around the film tip. This demonstrates that *V* not only possesses higher signal-to-noise ratio but also comprises of more comprehensive information of the flow dynamic, and is more responsive towards small-scale turbulence in comparison with camera recorded *δ*.

**Figure 13:**
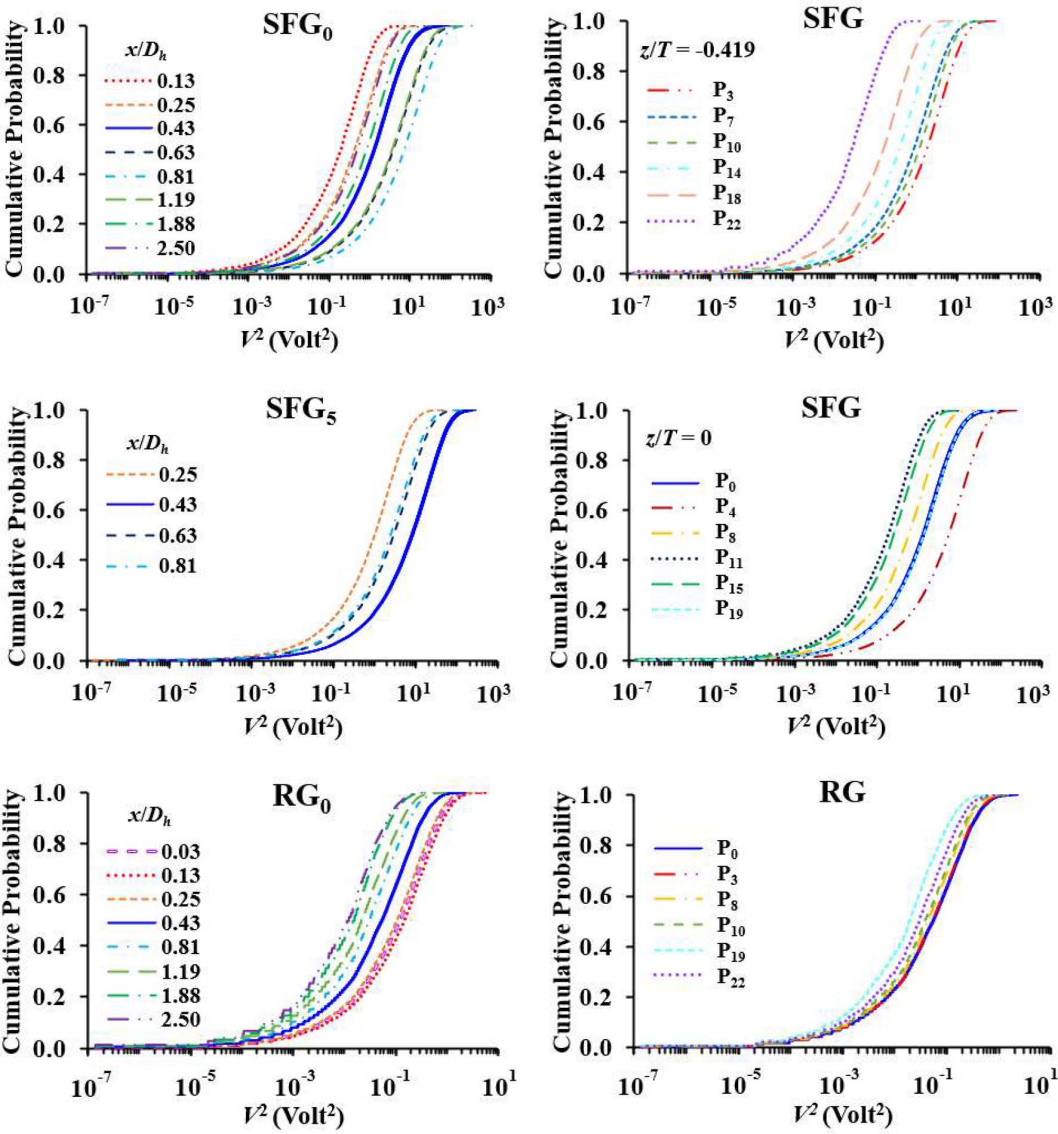
Cumulative probability of *V*^*2*^ at different streamwise locations along SFG(*P*_0_, *P*_5_) and RG centreline (left), and at *x*/*D*_*h*_ = 0.425 downstream from the SFG and RG (right).

Considering that *V* better characterizes grid-induced turbulence than the camera recorded *δ*, we attempt to examine the probability distribution of a random piezoelectric voltage response towards the turbulent flows generated by SFG and RG. On the left side of Fig. 13, we present the cumulative probability of *V*^2^ at different *x*/*D*_*h*_ for SFG_0_, SFG_5_ and RG_0_. It can be observed that the variations in the probability distribution with *x*/*D*_*h*_ for all three cases follow the streamwise profiles in Fig. 5a,c. For SFG_0_, at *x*/*D*_*h*_ = 0.13 where *V*_*rms*_, *δ*_*rms*_ and *I*_*y*_ are the lowest, the probability in obtaining large *V*^2^ is also the lowest, of which, 99% are less than 3 V^2^. At *x*_*peak*_/*D*_*h*_ = 0.81, 95% of *V*^2^ are below 68 V^2^ while the top 1 percentile has *V*^2^ > 120 V^2^, which is highly similar to the probability distribution at *x*_*peak*_/*D*_*h*_ = 0.43 of SFG_5_. On the contrary, RG_0_ has much smaller *V*^2^ as compared to SFG(P_0_, P_5_). For the former, 99% of the *V*^2^ at *x*_*peak*_/*D*_*h*_ = 0.13 do not even exceed 3 V^2^ and this is further reduced to 0.2 V^2^ at *x*/*D*_*h*_ = 2.50.

On the right of Fig. 13, we plot the cumulative probability of *V*^2^ at different lateral positions of SFG and RG where *x*/*D*_*h*_ = 0.425. It is immediately apparent that the variations in the distribution plots from middle to the bottom wall of test section for both grids are once again identical to the *V*_*rms*_ profile in Fig. 9c,g, with RG having a narrower range of variation than the SFG. For the latter, the probability in obtaining large *V*^2^ is high on P_3_ and P_4_, where top 5 percentile have *V*^2^ > 16 and 47 V^2^ while top 1 percentile have *V*^2^ > 30 and 88 V^2^, respectively. In the wake of the largest grid bar (P_8_ and P_11_), 99% of *V*^2^ are not more than 6 V^2^ on average. *V*^2^ is observed to be the lowest on P_22_ where 99% are below 0.5 V^2^, which is similar to P_22_ of RG. For the rest of the positions, lower *V*^2^ are attained for RG in comparison with SFG. It is also more likely to obtain small *V*^2^ closer to the bottom wall for instance, top 1 percentile of RG(P_0_, P_3_) has *V*^2^ above 0.8 V^2^ but is narrowed down to 0.3 V^2^ on P_19_.

In short, Fig. 13 not only demonstrates the dependency of voltage response on the grid-film distance and film lateral placement in the lee of grid, but also the unique advantages of SFG-induced turbulence over the turbulence generated by RG. Hence, this crucial finding presents opportunity for future study on correlating the piezoelectric output voltage with the various turbulence statistics and forcing of fractal grid-generated turbulence to unveil the multi-scale turbulence-mechanical interplays of fractal grid.

Furthermore, the overall high voltage response secured from the SFG-induced turbulence presents potential future energy harvesting opportunities. Piezoelectric thin-films could be placed at the regions where local turbulence strength is high to maximize the amount of electrical power harvested, particularly at the four P_5_ locations leeward of the SFG. Further research may be undertaken to scale-up the energy harnessing capability of the system, which include but not limited to optimising the geometrical parameters of the SFG and materials properties of the piezoelectric thin-films. Such energy harvesting module which consists of a SFG and an array of piezoelectric cantilever beams could be installed in various sections of the heating, ventilation and air conditioning (HVAC) ducts. The micro to milliwatt-scale electrical output harnessed may be used for self-powering low-power temperature and humidity sensors to monitor, control, and manage the HVAC systems. At the same time, the piezoelectric thin-films could also act as a flow sensing device to characterise the flow in the air ducts which could not be achieved by small wind turbines or solar panels.

## Conclusion

The present experimental study was set out to explore the mechanical characteristics of fractal grid-generated turbulence based upon the direct fluid–structure interaction between the flow and a flexible piezoelectric thin-film. The film undulation *δ* and voltage response *V* at different grid-film distances uniquely revealed the strength and the corresponding coverage of turbulence generation and decay regimes. Interestingly, the wake-interaction at P_5_ not only shifts the peak location *x*_*peak*_ upstream but also enhances the local turbulence strength generated using SFG. Centerline results at *x*_*peak*_ showed that the *V*_*rms*_ and *δ*_*rms*_ in the lee of SFG are 7× larger than that of the RG of equivalent blockage ratio *σ*, with the former having a millinewton turbulence forcing *F*_*rms*_ that is more than twice of RG.

The cross-sectional profiles of the thin-film’s physical response at *x*/*D*_*h*_ = 0.425 disclosed the inhomogeneity of fractal grid-generated flow in the turbulence generation region as compared to the relatively uniform flow induced by RG. The equivalent lateral integral length scale *L*_*v*_ of the vortices shed from the multi-scale SFG bars ranges from 8.7 to 12.8 mm which on average is 1.3× larger than RG. Low frequency, large-scale energy-containing eddies at the central opening of SFG are primarily responsible for the high voltage generation, of which, the average cross-sectional *V*_*rms*_ and *F*_*rms*_ are respectively, 5× and 2× higher than RG, with SFG_5_ having the highest values. Nevertheless, the recirculating flow in the wake of the largest grid bar, in addition to the highly chaotic flow stirred up by the small-scale eddies broken up by the corner walls of test section impose thin-film damping upon fractal grid-generated turbulence, which in turn weaken the local vortex shedding effect.

Our findings demonstrate the unique expression of insert-induced turbulence mechanical characteristics via PTFV, along with the characterization of the large-size flow structures’ turbulence length scale desired for effective thermal dissipation and energy harvesting. Such system could potentially be employed in real-world applications for the purpose of evaluating the flow’s local turbulence strength to gain insights into the broad-band random forcing across turbulence of various scales.

## References

[1] Verbeek AA, Bouten TWFM, Stoffels GGM, Geurts BJ, van der Meer TH (2015). Fractal turbulence enhancing low-swirl combustion. Combust. Flame.

[2] Teh AL (2015). Thermal mixing enhancement of a free-cooling system with a fractal orifice plate. Chem. Eng. Res. Des..

[3] Skanthan S, Yeoh CV, Chin WM, Foo JJ (2018). Forced convective heat transfer and flow characteristics of fractal grid heat sinks. Int. J. Therm. Sci..

[4] Hoi SM, Teh AL, Ooi EH, Chew IML, Foo JJ (2019). Forced convective heat transfer optimization of plate-fin heat sink with insert-induced turbulence. Appl. Therm. Eng..

[5] Mandelbrot BB (1983). The Fractal Geometry of Nature.

[6] Kochergin V, Kearney M (2006). Existing biorefinery operations that benefit from fractal-based process intensification. Appl. Biochem. Biotech..

[7] Laizet S, Vassilicos JC (2011). DNS of fractal-generated turbulence. Flow Turbul. Combust..

[8] *Piezo film sensors technical manual*. 2-3 (Measurement Specialties, Inc., 1999).

[9] Akaydin HD, Elvin N, Andreopoulos Y (2010). Wake of a cylinder: A paradigm for energy harvesting with piezoelectric materials. Exp. Fluids.

[10] Goushcha O, Akaydin HD, Elvin N, Andreopoulos Y (2015). Energy harvesting prospects in turbulent boundary layers by using piezoelectric transduction. J. Fluids Struct..

[11] Danesh-Yazdi AH, Goushcha O, Elvin N, Andreopoulos Y (2015). Fluidic energy harvesting beams in grid turbulence. Exp. Fluids.

[12] Meng J, Xie W, Brennan M, Runge K, Bradshaw D (2014). Measuring turbulence in a flotation cell using the piezoelectric sensor. Miner. Eng..

[13] Tabosa, E., Runge, K. & Holtham, P.N. Development and application of a technique for evaluating turbulence in a flotation cell. In *XXVI International Mineral Processing Congress-IMPC 2012* 5377–5390 (Technowrites, 2012).

[14] Akaydin HD, Elvin N, Andreopoulos Y (2010). Energy harvesting from highly unsteady fluid flows using piezoelectric materials. J. Intell. Mater. Syst. Struct..

[15] Akaydin HD, Elvin N, Andreopoulos Y (2012). The performance of a self-excited fluidic energy harvester. Smart Mater. Struct..

[16] Danesh-Yazdi AH, Elvin N, Andreopoulos Y (2016). Parametric analysis of fluidic energy harvesters in grid turbulence. J. Intell. Mater. Syst. Struct..

[17] Ferko, K., Lachendro, D., Bradley, A. & Danesh-Yazdi, A.H. Feasibility study of interacting side-by-side piezoelectric harvesters in low-intensity grid-generated turbulence. In *Active and Passive Smart Structures and Integrated Systems XII* (ed. Erturk, A.) 105950Q (SPIE, 2018).

[18] Ferko, K., Chiappazzi, N., Gong, J. & Danesh-Yazdi, A.H. Average power output and the power law: Identifying trends in the behavior of fluidic energy harvesters in grid turbulence. In *Active and Passive Smart Structures and Integrated Systems XIII* (ed. Erturk, A.) 109670Z (SPIE, 2019).

[19] Ferko, K., Lachendro, D., Chiappazzi, N. & Danesh-Yazdi, A.H. Interaction of side-by-side fluidic harvesters in fractal grid-generated turbulence. In *Active and Passive Smart Structures and Integrated Systems XII* (ed. Erturk, A.) 105951E (SPIE, 2018).

[20] Ferko, K., Chiappazzi, N., Gong, J. & Danesh-Yazdi, A.H. Power output comparison of side-by-side fluidic harvesters in different types of fractal grid-generated turbulence. In *Active and Passive Smart Structures and Integrated Systems XIII* (ed. Erturk, A.) 109670P (SPIE, 2019).

[21] Mazellier N, Vassilicos JC (2010). Turbulence without Richardson-Kolmogorov cascade. Phys. Fluids.

[22] *LDT1-028K piezo sensor w/ leads attached*. (Measurement Specialties, Inc., 2015).

[23] Munson BR, Young DF, Okiishi TH, Huebsch WW (2009). Fundamentals of Fluid Mechanics.

[24] Sodano HA, Park G, Inman DJ (2004). Estimation of electric charge output for piezoelectric energy harvesting. Strain.

[25] Elvin NG, Lajnef N, Elvin AA (2006). Feasibility of structural monitoring with vibration powered sensors. Smart Mater. Struct..

[26] Casiano, M.J. *Extracting damping ratio from dynamic data and numerical solutions*. 2–4 (2016). At https://ntrs.nasa.gov/citations/20170005173

[27] Hobeck JD, Inman DJ (2012). Artificial piezoelectric grass for energy harvesting from turbulence-induced vibration. Smart Mater. Struct..

[28] Inman DJ (2013). Engineering Vibration PIE.

[29] Elvin NG, Elvin AA (2008). A coupled finite element—circuit simulation model for analyzing piezoelectric energy generators. J. Intell. Mater. Syst. Struct..

[30] Hurst D, Vassilicos JC (2007). Scalings and decay of fractal-generated turbulence. Phys. Fluids.

[31] Maxey MR (1987). The velocity skewness measured in grid turbulence. Phys. Fluids.

[32] Zhou Y (2014). Relevance of turbulence behind the single square grid to turbulence generated by regular- and multiscale-grids. Phys. Fluids.

[33] Melina G, Bruce PJK, Vassilicos JC (2016). Vortex shedding effects in grid-generated turbulence. Phys. Rev. Fluids.

[34] Gomes-Fernandes R, Ganapathisubramani B, Vassilicos JC (2015). The energy cascade in near-field non-homogeneous non-isotropic turbulence. J. Fluid Mech..

[35] Nagata K, Saiki T, Sakai Y, Ito Y, Iwano K (2017). Effects of grid geometry on non-equilibrium dissipation in grid turbulence. Phys. Fluids.

[36] Nagata K (2013). Turbulence structure and turbulence kinetic energy transport in multiscale/fractal-generated turbulence. Phys. Fluids.

[37] Blevins RD (2001). Flow-Induced Vibration.

